# Quick returns, sleep, sleepiness and stress – An intra-individual field study on objective sleep and diary data

**DOI:** 10.5271/sjweh.4175

**Published:** 2024-09-01

**Authors:** Kristin Öster, Philip Tucker, Marie Söderström, Anna Dahlgren

**Affiliations:** 1 The division of Psychology, Department of Clinical Neuroscience, Karolinska Institute, Solna, Sweden.; 2 Stress Research Institute, Department of Psychology, Stockholm University, Stockholm, Sweden.; 3 School of Psychology, Swansea University, Swansea, UK.

**Keywords:** actigraphy, backward rotation, fatigue, recovery, safety, shift work, work schedule tolerance

## Abstract

**Objectives:**

Quick returns (<11 hours of rest between shifts) have been associated with shortened sleep length and increased sleepiness, but previous efforts have failed to find effects on sleep quality or stress. A shortcoming of most previous research has been the reliance on subjective measures of sleep. The aim of this study was to combine diary and actigraphy data to investigate intra-individual differences in sleep length, sleep quality, sleepiness, and stress during quick returns compared to day-day transitions.

**Methods:**

Of 225 nurses and assistant nurses who wore actigraphy wristbands and kept a diary of work and sleep for seven days, a subsample of 90 individuals with one observation of both a quick return and a control condition (day-day transition) was extracted. Sleep quality was assessed with actigraphy data on sleep fragmentation and subjective ratings of perceived sleep quality. Stress and sleepiness levels were rated every third hour throughout the day. Shifts were identified from self-reported working hours. Data was analyzed in multilevel models.

**Results:**

Quick returns were associated with 1 hour shorter sleep length [95% confidence interval (CI) -1.23– -0.81], reduced subjective sleep quality (-0.49, 95% CI -0.69– -0.31), increased anxiety at bedtime (-0.38, 95% CI -0.69– -0.08) and increased worktime sleepiness (0.45, 95%CI 0.22– 0.71), compared to day-day transitions. Sleep fragmentation and stress ratings did not differ between conditions.

**Conclusions:**

The findings of impaired sleep and increased sleepiness highlight the need for caution when scheduling shift combinations with quick returns.

Two in ten European workers are exposed to short rest periods (<11 hours) between shifts, most commonly within healthcare, agriculture, construction, and transport (1). The most common quick return is an evening shift followed by a day shift (2), which is the focus of the present paper. In Nordic healthcare, there has been a high prevalence of quick returns, despite their proscription by European legislation (3), although recent alterations of regulations in Sweden and Finland might change this. Between 20–68% of nurses are estimated to be exposed to quick returns (4–6).

Quick returns compress work hours, allowing for longer consecutive periods off work, which is often valued by shift workers (7). Within 24-hour healthcare settings, working the evening before a day shift may also promote continuity in work processes (8) thereby potentially increasing control and alleviating stress during the morning shift. However, quick returns also restrict the time available for sleep and recovery between shifts.

Quick returns are associated with both short sleep length (≤6.5 hours) (2, 9) and reduced sleep quality in general (2, 4). As sleep is important for performance (10, 11), quick returns are potential risk factors for safety and, in the longer term, also for health (see figure 1). In addition, quick returns are associated with difficulties unwinding (8), worry, and difficulties detaching from work (12), which could contribute to difficulties falling asleep or affect sleep quality negatively. While previous studies failed to detect acute effects on sleep quality compared to other shift transitions (9, 13, 14), they mainly relied on self-reported measurements of sleep which can be unreliable.

**Figure 1:**
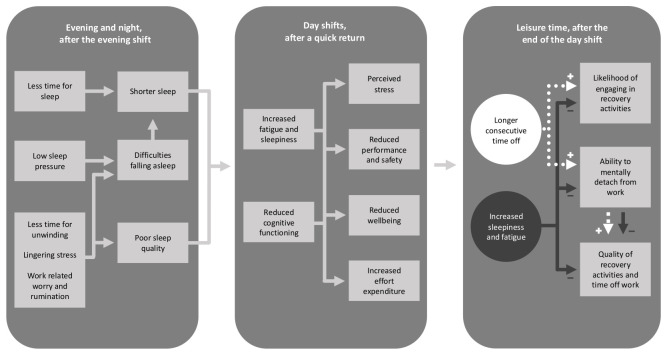
Suggested mechanisms through which quick returns between evening and day shifts may affect sleep, and thereby contribute to fatigue, stress and reduced performance and wellbeing during the day shift, and subsequently affect the quality of leisure time after work, and thereby counteract some of the positive effects of having more consecutive time off work.

As a consequence of short sleep, quick returns are associated with increased daytime sleepiness (2, 9) and fatigue (2, 13, 15). It is unclear, however, if quick returns increase sleepiness throughout the day or cause elevated sleepiness during certain time periods during the day. One study reported higher sleepiness in the beginning of morning shifts that were preceded by a quick return (14), but qualitative data indicate that the most severe fatigue may come after work hours (12). Understanding if and how sleepiness varies across the workday as a consequence of quick returns is important for successful fatigue risk management, for example in the planning of safety critical work tasks.

As well as sleep, recovery activities are also important for health and wellbeing (16, 17). After a quick return, workers usually gain longer consecutive periods off work, which are likely to promote recovery. However, quick returns have also been associated with increased fatigue during free time (8, 13, 15) and difficulties detaching from work (12). As a consequence, workers may be too tired to engage in leisure-time activities despite having more spare time, and the quality of recovery activities may be reduced following a quick return (17), as indicated on the right of figure 1.

Quick returns have been associated with impaired health and wellbeing (2), increased stress (2, 4, 9), prospective sick leave (18), accidents, and mistakes (5, 19, 20). It is likely that many of these consequences derive from insufficient sleep and recovery (11). For example, stress activation may occur on the second day of a quick return as a compensatory measure to cope with increased fatigue (21). Interventions involving a change from backward to forward rotation (eg, changing the sequence of shifts from evening-to-day shift transitions to day-to-evening transitions), thereby removing quick returns, are among the most promising working hour interventions for the promotion of sleep and health (22).

In sum, many studies provide reliable evidence that quick returns shorten sleep and result in fatigue. However, previous studies have failed to find acute effects on sleep quality (9, 13) and stress (9). Moreover, there is a paucity of objective data on length and quality of sleep that would enable more precise estimates. Objective measures of sleep in relation to quick returns would increase both the evidence base and our understanding of possible mechanisms through which quick returns may affect health and safety outcomes.

The aim of this study was to combine subjective (diary) and objective (actigraphy) data to investigate intra-individual differences in sleep length, sleep quality, sleepiness and stress during quick returns compared to day-day transitions in a sample of nurses and assistant nurses. During the night of a quick return, we hypothesized that sleep length would be shortened (Hypothesis 1) and that both subjective (sleep quality index) and objective (sleep fragmentation) measures of sleep quality would be reduced (Hypothesis 2). On the second day of a quick return, we expected to find increased sleepiness during work (Hypothesis 3) and leisure time (Hypothesis 4), and increased stress during work (Hypothesis 5). Lastly, we predicted that there would be an interaction between shift sequence (ie, either day-day or a quick return) and time of day in sleepiness ratings, so that sleepiness would be higher toward the end of the second shift after a quick return (Hypothesis 6). We also aimed to test our hypotheses of reduced sleep quality and increased stress on secondary outcomes and expected to find (i) feelings of increased anxiousness at bedtime, (ii) feeling less rested in the morning, (iii) lower sleep efficiency, (iv) impaired psychological detachment, and (v) feeling more tense in relation to quick returns.

## Methods

### Study design and population

The present intra-individual study was based on diary and actigraphy data, provided by nurses and assistant nurses during seven consecutive days. Data was retrieved from the baseline survey of two independent intervention studies. Each participant acted as their own control, where data from a quick return and a day-day transition was compared for every individual.

The first intervention study investigated the effects of a recovery program directed at newly graduated nurses (inclusion criteria was <12 months of work experience) during 2017–2018. Out of 461 potentially eligible invited participants, 207 completed the baseline survey and were included.

The second study investigated the effects of a work time intervention aimed at reducing the number of quick returns among nurses and assistant nurses. The baseline survey took place in the fall of 2019. A total of 366 employees at the participating wards received information about the study (the number of invitees who were potentially eligible to participate, ie, who met the inclusion criteria of having a schedule with quick returns, is unknown), of whom 97 completed the survey and 96 were eligible for participation. One participant was excluded due to working permanent night shifts.

The participants worked at different 24-hour care units at different hospitals in Sweden that spanned a representative range of patients and type of care, for example, emergency wards, palliative wards, geriatrics, pediatrics, oncology, abdominal surgeries and psychiatry care units. A full description of the designs and first results of both studies have been previously reported (8, 23). All participants gave their informed consent prior to participate in the studies.

The participants could choose to take part in an intensified investigation in addition to the baseline survey. This involved wearing an actigraphy wristband during the sleep periods (CamNtech Ltd, United Kingdom) and keeping a diary of work and sleep for seven consecutive days at the baseline and post-intervention measurements. The participants were asked to start recording their week after a day off work. In the second study, participants were asked to pick a week when they were scheduled to work a quick return. The diary and actigraphy data collected at baseline were used for the purpose of the current study.

In total, 225 participants from the two studies took part in the intensified investigation at baseline. The analysis included those with at least one observation of both a quick return and a day-day transition, resulting in a final sample of 90 individuals.

### Assessment of exposure

Shifts were identified from self-reported work hours. Quick returns were defined as having <11 hours of rest between two shifts. Control conditions were defined as day-day transitions.

Evening shifts were defined as shift durations of ≥3 hours that ended after 20:00 and were not night shifts. Night shifts were defined as ≥ 3 hours of work between 23:00–05:00. Day shifts were defined as all other shifts of at least 3 hours. The cut off for evening shifts at >20:00 deviates from that used in previous research. The decision was based on the actual working hours observed in the sample: some workers who started at 12:00 or later left work as early as 20:00, whereas all participants who started work before noon went off their shift prior to 20:00.

All quick returns (<11 hours of rest between shifts) identified in the sample happened to take place between an evening (average work hours 13:30–21:42) and day shift (average work hours 06:54–15:42). In two cases, the second shift of the quick return was a double shift (≈07:00–21:30). In the analysis, we compared the first and second day of quick returns to the first and second day of a control condition (day-day transitions).

### Outcomes

Primary and secondary outcomes, and operationalizations of variables were pre-registered (https://osf.io/d3eaq) prior to analyzing data.

### Primary outcomes

Stress was assessed on a likert scale ranging from 1 (“very low stress, feeling very relaxed and calm”) to 9 (“very high stress, feeling tense and pressured – at the limit of my capabilities”). The stress rating scale was inspired by the validated Stress–Energy Rating Questionnaire (24), which has been adapted for collecting data several times daily (25). Sleepiness (1=extremely alert, 9=extremely sleepy, fighting sleep) was assessed with the Karolinska sleepiness scale (KSS) (26).

Both stress and sleepiness were rated continuously during the day, every third hour from 07:00. Stress and sleepiness during work hours were operationalized as the ratings during work hours or within 15 minutes of having started or ended one’s shift. This operationalization maximized the use of data and still ensured the validity of the outcome measures. As sleepiness is influenced by circadian rhythms, the ratings of sleepiness at work and after work (ie, leisure time) were analyzed in relation to a day shift (after a quick return or no quick return). Thus, sleepiness during work was extracted from work hours between 07:00–16:00 (ie, the typical hours of a day shift) and sleepiness during leisure time was extracted from leisure time ratings between 16:00–22:00.

Sleep quality was assessed with both actigraphy data on sleep fragmentation (Sleep Fragmentation Index, which is the sum of the mobile time (%) and the immobile bouts ≤1 minute (%) and the Karolinska sleep diary – sleep quality index (KSD-SQI, 1=poor, 5=good) (27).

Sleep length was measured with actigraphy data, and was operationalized as the total elapsed time between falling asleep and waking up.

### Secondary outcomes

Anxiousness at bedtime on the night between the two shifts (1=very, 5=not at all) and feeling rested at waketime (1=not at all, 5=completely) were assessed using items from the KSD. Sleep efficiency was assessed using actigraphy data on actual sleep percentage (percentage of time spent asleep between the time of falling asleep and wake-up time). The item “difficulty letting go of work related thoughts during leisure time” (1=not at all, 5=to a large extent) was used to assess psychological detachment during leisure time in relation to both the first and second shift. The item “feeling tense throughout the day” (1=not at all, 5=to a large extent) was used as a secondary measure of stress during the second shift.

### Statistical analyses

The analysis plan was pre-registered prior to analysis in the OSF Registries (https://osf.io/d3eaq). As a starting point, all primary outcomes were analyzed in multilevel linear regression models with the maximum random effects structure justified by the design (28). For the outcomes stress and sleepiness, time of ratings was added as a covariate. Convergence problems were primarily addressed with numerical optimization procedures. Secondly, by-participant random slopes and random intercepts were removed one at a time, keeping the random slope for the main effect of interest. If a model failed to converge despite optimization and simplification, data were aggregated and analyzed with a paired t-test. See table 1 for the final model specifications. Secondary outcomes were analyzed with paired t-tests.

**Table 1:** Final model equations and random effects structures for the multilevel models. [β = fixed effects; μ = random effects; ε = residual error.]

Outcome	**Equation**
Fragmentation index	$Fragmentation=\beta_{0}+\beta_{1}\times Shift+\beta_{2}\times Nr.Workdays+\mu_{0}+\mu_{1}\times Shift+\epsilon$
Sleep quality index	Sleep quality index=β_0+β_1×Shift+β_2×Nr.Workdays+μ_0+μ_1×Shift+ϵ
Stress at work	$Stress=\beta_{0}+\beta_{1}\times Shift+\beta_{2}\times Nr.Workdays+\beta_{3}\times Time+\mu_{0}+\mu_{1}\times Shift+\mu_{2}\times Nr.Workdays+\epsilon$
Sleepiness at work	$Sleepiness=\beta_{0}+\beta_{1}\times Shift+\beta_{2}\times Nr.Workdays+\beta_{3}\times Time+\mu_{0}+\mu_{1}\times Shift+\mu_{2}\times Nr.Workdays+\epsilon$
Sleepiness after work	$Sleepiness=\beta_{0}+\beta_{1}\times Shift+\beta_{2}\times Nr.Workdays+\beta_{3}\times Time+\mu_{0}+\mu_{1}\times Shift+\epsilon$
Sleepiness at work – with interaction term	$Sleepiness=\beta_{0}+\beta_{1}\times Shift\times Time+\beta_{2}\times Shift+\beta_{3}\times Time+\beta_{4}\times Nr.Workdays+\mu_{0}+\mu_{1}\times Shift+\epsilon$

The decision to fit multilevel models to all primary outcomes deviates from our pre-registered plan and was motivated by the presence of order effects in the data: ≥41 (46%) participants had worked a combination of evening-day-day. Thus, lingering effects from quick returns could have spilled over onto a non-negligible proportion of the control conditions (day-day). To control for order effects, we added the number of prior workdays as a covariate. As potential interactions or nonlinear order effects could not be controlled for, we also performed sensitivity analyses in the form of unpaired t-tests using data from the second measurement day only. The sample size of the sensitivity analyses was smaller (N=72) and unbalanced (N=46 – quick return, N=26 – day-day transition) and should be interpreted accordingly. Sensitivity analyses were also performed to confirm the operationalizations of stress and sleepiness during work.

We adhered to the standard alpha level of 0.05 and two-tailed test. P-values were obtained by likelihood ratio tests comparing models with and without the effect of interest. Cohen’s d effect sizes were calculated by dividing the difference between the estimated means by the square root of the summarized variance of the random effects (29).

The raw output from the actigraphy sleep recordings were analyzed in Motionware 1.2.25–28, and were cross-checked against the participants’ diary recordings. All statistical analyses were conducted in R version 4.1.3. The R package lme4 1.1-31 (30) was used to fit multilevel models.

## Results

The sample mean age was 29.4 [standard deviation (SD) 7.7] years, 91% were women, 89% worked as nurses, 92% worked full time and no participants worked <75%. The mean experience within the profession ranged from 1–34 years (mean=3, SD=6.1). The average length of rest time during quick returns was 9 hours and 11 minutes (SD=31 minutes), and typically took place between 21:42 (SD=21 minutes) and 06:54 (SD=28 minutes).

Raw means and SD for the comparisons between shift sequences are presented in table 2, together with model estimates. Quick returns shortened sleep length by 1.02 hours [95% confidence interval (CI) -1.23– -0.81] compared to day-day transitions. There was no significant difference in fragmentation index. Subjective sleep quality, KSD-SQI, was reduced by a half scale point (-0.49, 95% CI -0.69– -0.31). Participants were sleepier both during work (0.45, 95% CI 0.22– 0.71) and leisure time (0.36, 95% CI 0.07– 0.59) on the second day of a quick return compared to the second day of a day-day transition. Worktime stress ratings did not differ significantly between conditions. During quick returns, participants rated that they were slightly (-0.38, 95% CI -0.69– -0.08) more anxious at bedtime and rated themselves half a scale point less well rested (-0.54, 95% CI -0.77– -0.31) when waking up. Quick returns were associated with a small decrease of sleep efficiency (-0.78, 95% CI -1.44– -0.11). There was no effect of quick returns for either psychological detachment or feeling tense.

**Table 2:** Raw means, standard deviations (SD), estimated differences and test statistics for all outcomes but the interaction effect. **Boldface denotes** a statistically significant effect (P<0.05). [CI=confidence interval.]

Outcomes (scale limits)		N ^c^	Day-day			Quick return			Estimated difference	t ^a^	χ^2^(1)^b^	P-value	Cohen’s d
			Mean	SD		Mean	SD		95% CI				
Primary outcomes, sleep between shifts													
	Sleep length (hours)	86	6.98	0.98		5.95	0.70		**-1.02 (-1.23– -0.81)**	-9.69		0.000	-0.26
	Fragmentation index (0–100)	87	19.75	8.87		22.71	9.39		1.72 (-0.26–3.82)		2.66	0.103	0.17
	KSD-SQI (1–5)	90	4.16	0.70		3.67	0.89		**-0.49 (-0.69– -0.31)**		20.79	0.000	-0.56
Primary outcomes, ratings during the day													
	Worktime sleepiness, day 2 (1–9)	90	4.51	1.76		4.80	1.69		**0.45 (0.22–0.71)**		10.77	0.001	0.20
	Off work sleepiness, day 2 (1–9)	90	5.63	2.00		5.89	1.92		**0.36 (0.07–0.59)**		4.61	0.032	0.18
	Worktime stress, day 2(1–9)	89	4.49	1.98		4.58	1.89		0.26 (-0.12–0.61)		2.12	0.146	0.11
Secondary outcomes, sleep between shifts													
	Anxious at bedtime (1–5)	90	3.92	1.20		3.56	1.31		**-0.38 (-0.69– -0.08)**	-2.47		0.015	-0.15
	Feeling rested (1–5)	90	2.55	1.03		1.99	0.90		**-0.54 (-0.77– -0.31)**	-4.60		0.000	-0.30
	Sleep efficiency (%)	87	89.63	3.96		88.73	4.26		**-0.78 (-1.44– -0.11)**	-2.31		0.024	-0.02
Secondary outcomes, ratings of the day													
	Psychological detachment, day 1 (1–5)	90	2.28	1.37		2.39	1.33		0.22 (-0.06–0.50)	1.54		0.127	0.16
	Psychological detachment, day 2 (1–5)	90	2.17	1.32		2.35	1.38		0.21 (-0.06–0.47)	1.57		0.120	0.12
	Feeling tense, day 2 (1–5)	90	2.33	1.29		2.39	1.26		0.02 (-0.27–0.31)	0.11		0.909	0.01

^a^ t-statistics are derived from outcomes analyzed with paired t.tests. ^b^ χ^2^(df) statistics are derived from the multilevel models. These estimates are adjusted for order effects, operationalized as the number of consecutive workdays, and time when applicable. See table 1 for the full model specification. ^c^ Number of participants with complete data for the variable of interest, and thus included in the analysis.

**Figure 2:**
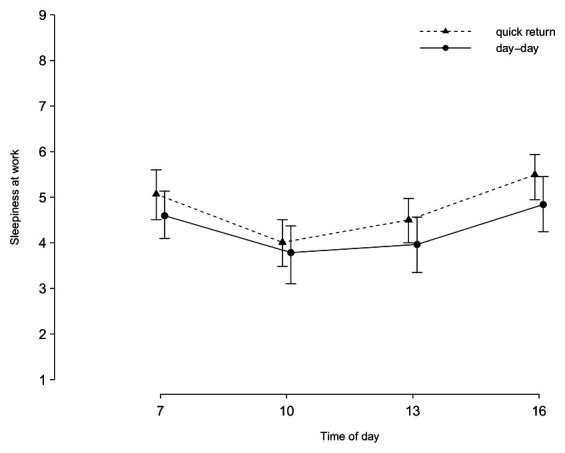
Estimated sleepiness during quick returns and day-day transitions. Error bars display the confidence interval.

Raw means and SD of stress and sleepiness rating across the second shift of both shift sequences are presented in table 3. The effect of quick returns on sleepiness did not interact significantly with time [$X^{2}$ (1)=2.46, P=0.483], indicating that the difference in sleepiness between shift conditions did not vary significantly across the workday (see figure 2).

**Table 3:** Raw means and standard deviation (SD) for stress and sleepiness during the second shift, across time points.

Time of day (hour)	Sleepiness						Stress				
	Day-day			Quick return			Day-day			Quick return	
	Mean	SD		Mean	SD		Mean	SD		Mean	SD
07:00	5.01	1.84		5.23	1.72		4.01	1.85		4.21	1.66
10:00	4.11	1.55		4.23	1.49		4.55	2.05		4.64	1.71
13:00	4.30	1.67		4.66	1.60		4.67	2.03		5.02	2.06
16:00	5.12	1.87		5.70	1.84		4.90	1.83		4.20	2.18

The sensitivity test of operationalizations indicated that the cut-offs used for stress and sleepiness at work did not impose bias on the results. The sensitivity tests of order effects resulted in differences that were close to and in the same direction as most of the significant model estimates, with three exceptions. Regarding sleep efficiency and fragmentation index, the sensitivity analysis resulted in observed differences (1.26 and -2.04, respectively) in the opposite direction compared to the main analysis. When fragmentation index was analyzed with a paired t-test, in accordance with our pre-registration, the observed difference between conditions remained small (2.3, P=0.024) but was significant. For sleepiness during leisure, the estimated difference was reduced by half in the sensitivity analysis (difference=0.18). For the full model output and detailed results of the sensitivity analyses, see the supplementary material (www.sjweh.fi/article/4175).

## Discussion

In line with our hypotheses, quick returns were associated with one hour shorter sleep length (H1), decreased subjective sleep quality (H2), and increased work- and leisure-time sleepiness compared to day-day transitions (H3 and H4). Contrary to our expectations, there was no significant interaction between shift and time of day, when analyzing sleepiness (H6). Quick returns did not affect stress levels (H5) nor objective measures of sleep quality (H2).

In accordance with most previous studies (2, 9), quick returns were found to shorten sleep length. With an average sleep length of 6 hours, quick returns clearly impede sleep. In epidemiological studies, a sleep length of ≤6 hours is associated with an increased risk of all-cause mortality (31).

Regarding sleep quality, the results are less conclusive. Contrary to our hypothesis, we found no significant difference in fragmentation index across conditions and only a small decrease of sleep efficiency in the main analysis. In the additional sensitivity tests, these results could not be verified, which indicates that the finding of decreased sleep efficiency could be due to chance or confounding. Contrary to previous studies that failed to find a difference in subjective measures of sleep quality during quick returns (9, 13, 14), our results showed reduced subjective sleep quality and increased anxiousness at bedtime. Two of the previous studies used a single item to measure sleep quality, either in terms of restless sleep or the perception of how well one has slept. The Karolinska sleep quality index, used in this and one previous study (14), is a composite of items assessing ease of falling asleep, sleep quality, calm sleep and premature awakenings. Objective data from both the current and previous study (which compared sleep on quick returns with sleeps after rest days; 14) failed to indicate an increase in restless sleep during a quick return. Thus, it seems possible that the observed difference in subjective sleep quality in this study captures other aspects of sleep quality. More studies combining objective data with validated measures of subjective sleep quality are needed to determine if there is a negative effect of quick returns on sleep quality.

Nurses and assistant nurses were found to feel less rested and more sleepy after quick returns compared to after day-day transitions, indicating that sleep and recovery during quick returns is not only reduced but insufficient. The magnitude of both these differences was half a scale step, which is in line with previous estimates (9).

Looking at the mean sleepiness ratings, our data indicate that sleepiness is already a challenge for nurses working day-day transitions and is further aggravated during quick returns. Sleepiness scores of ≥7 have been associated with itchy eyes, changes in EEG patterns and severe lapses in attention (32). Thus, the observed average score close to 5 (“neither alert nor sleepy”) is approaching levels of sleepiness that negatively impact work performance. Although most nurses working quick returns did not run the risk of dozing off, the underlying sleepiness and reduced alertness may have affected their ability to interact with patients and perform complex cognitive and safety critical tasks. Moreover, there was large individual variation, where some nurses did report alarmingly high levels of sleepiness. In addition, the observed differences in sleepiness may be an underestimate due to the presence of order effects and spillover fatigue, which could only be partially controlled for in the analysis.

The increased sleepiness during a quick return, relative to a day-day transition, was constant throughout the day. This was reflected in the absence of a significant interaction effect between shift sequence and time of day. As shown in figure 2, there was the expected u-shaped variation in sleepiness (32). Sleepiness ratings decreased around midday and increased again in the afternoon. These variations are likely a product of circadian rhythms (33), but it is also possible that high activity levels around midday suppress some sleepiness as stress and sleep are physiological counterparts (34). However, even if stress or increased activity may momentarily suppress sleepiness, it does not seem to counteract the overall increase in sleepiness observed in connection with a quick return.

Even though the observed difference in sleepiness was modest, the implications of increased sleepiness for health and safety are important considerations when planning shift schedules. Monotonous and time extended tasks are known to increase sleepiness, whereas rest breaks, light exposure and physical activity increase alertness (32). Thus, monitoring fatigue levels, planning for recurrent rest breaks, allowing for variation in work tasks and limiting overtime may be suitable solutions to prevent excessive sleepiness and reduced performance following quick returns. Moreover, participants were most sleepy during the early and late hours of the day shift. If possible, it may be wise to avoid the most safety critical work tasks during the beginning and end of the shift, or to buffer performance deficits (eg, double checking of safety critical procedures and providing support guidelines to staff).

Previous research has associated quick returns with increased fatigue during free time (8, 13, 15). This is in line with our results showing a small but significant difference in sleepiness between quick returns and day-day transitions during non-work hours. In the sensitivity test, the magnitude was halved. Contrary to previous qualitative evidence (12), we found no difference in ratings of detachment from work on either day of the respective shift sequences. Counteracting mechanisms could be present. On the one hand, limited time for unwinding after the first shift, and fatigue after the second shift, could both cause difficulties fending off worry and rumination. On the other hand, knowing that one does not have to return to work soon may also make it easier to disconnect mentally after the second shift. In relation to the first shift, nurses may benefit from the greater sense of continuity in work process that is sometimes associated with evening-to-morning shift transitions in healthcare [eg, knowing what to expect in the upcoming day shift as a result of having worked the preceding evening shift (8)]. Sleepiness and fatigue resulting from quick returns could theoretically inhibit active forms of recovery during leisure time (16), as is suggested in figure 1. However, it is not clear from our results that quick returns interfere more with active recovery during leisure time compared to day-day transitions.

Our results corroborate several previous findings from research on quick returns. Quick returns were found to shorten sleep length (2, 9), which was manifested in slightly heightened sleepiness during the second shift (9, 14, 35). As both the present study and a previous diary on nurses in Norway (9) identified associations between quick returns and both shortened sleep length and increased sleepiness, these findings are likely generalizable to other hospital settings. From a theoretical point of view, it also seems reasonable to assume that when time is limited for sleep, sleep is shortened; and when sleep is shortened, sleepiness increases. In line with previous research (9), quick returns did not cause more stress than day-day transitions, indicating that other factors are likely to be more important for the stress experience during work. While previous studies found no effect on subjective sleep quality (9, 13, 14), the present study did but this was not reflected in objective measures.

In sum, our findings support many but not all of the suggested mechanisms set out in figure 1. The present study gives support to the notion that quick returns contribute to increased anxiety at bedtime which may contribute to difficulties falling asleep, shortened sleep length and modestly increased sleepiness the following day both during work and non-work hours. There was no support for the notion that quick returns cause stress or affect psychological detachment, and mixed support for reduced sleep quality. Effects on wellbeing, cognitive functioning and performance, and leisure time quality were not assessed, but need to be evaluated in future studies using both objective and subjective data.

### Strengths and limitations

Strengths of the current study include the combination of objective and subjective measures and the intra-individual design. Self-reports can be unreliable, thus objective measures improve estimates of sleep length and fragmentation. The subjective experience of sleep quality and sufficiency are also important and were thoroughly assessed with several items from a validated questionnaire. The intraindividual design gave increased statistical power.

Limitations to the study include the presence of order effects. Almost half of the participants had worked the shift combination *evening-day-day*, where sleepiness from the quick return between the *evening-day* transition may have spilled over onto the third workday, the *day-day* transition. The analysis controlled for the number of consecutive workdays but did not control for potential nonlinear effects or interactions. In addition, as type of shift and the number of consecutive workdays were correlated, the analysis could not fully discern the effect of consecutive workdays from that of shift type. Thus, the results may be biased which could cause both under and overestimation of effects, depending on the outcome. The sensitivity analysis reported gives some indication of the validity of the magnitude and direction of these effect, but future studies are needed to draw more reliable conclusions. Another limitation is that we do not know how representative our samples are.

To minimize confounding, it would be desirable to compare shift combinations that were preceded by a day off work. Possible solutions are to follow more participants for longer time periods to increase the chances of finding such unbiased comparisons. Another is to adopt a quasi-experimental approach by asking participants to schedule the comparisons of interest.

### Concluding remarks

Quick returns impede recovery and result in increased sleepiness. Thus, frequent quick returns are likely to have negative acute effects on performance, and possibly also negative long-term effects on health. What constitutes a safe number of quick returns remains a question for future research. There is also a need for more research to understand individual differences in tolerance of quick returns.

## Supplementary material

Supplementary material

## References

[1] Eurofound. Sixth European Working Conditions Survey – Overview report (2017 update). Luxembourg: Publications Office of the European Union; 2017.

[2] Vedaa Ø, Harris A, Bjorvatn B, Waage S, Sivertsen B, Tucker P et al. Systematic review of the relationship between quick returns in rotating shift work and health-related outcomes. Ergonomics 2016;59(1):1–14. 10.1080/00140139.2015.1052020 26072668

[3] European Union. Directive 2003/88/EC of the European Parliament and of the Council of 4 November 2003 concerning certain aspects of the organisation of working time. Official Journal of the European Union. 2003;L299:9–19.

[4] Dahlgren A, Tucker P, Gustavsson P, Rudman A. Quick returns and night work as predictors of sleep quality, fatigue, work-family balance and satisfaction with work hours. Chronobiol Int 2016;33(6):759–67. 10.3109/07420528.2016.1167725 27082143

[5] Vedaa Ø, Harris A, Waage S, Bjorvatn B, Thun E, Buchvold HV et al. A longitudinal study on the association between quick returns and occupational accidents. Scand J Work Environ Health 2020 Nov;46(6):645–9. 10.5271/sjweh.3906 32632456 PMC7737807

[6] Vedaa Ø, Harris A, Erevik EK, Waage S, Bjorvatn B, Sivertsen B et al. Short rest between shifts (quick returns) and night work is associated with work-related accidents. Int Arch Occup Environ Health 2019 Aug;92(6):829–35. 10.1007/s00420-019-01421-8 30879132

[7] Nabe-Nielsen K, Lund H, Ajslev JZ, Hansen ÅM, Albertsen K, Hvid H et al. How do employees prioritise when they schedule their own shifts? Ergonomics 2013;56(8):1216–24. 10.1080/00140139.2013.815804 23826655

[8] Öster K, Tucker P, Söderström M, Dahlgren A. Pros and cons of quick returns-a cross-sectional survey among Swedish nurses and nurse assistants. Ind Health 2023 Sep;61(5):379–92. 10.2486/indhealth.2022-0033 35896350 PMC10542474

[9] Vedaa Ø, Mørland E, Larsen M, Harris A, Erevik E, Sivertsen B et al. Sleep Detriments Associated With Quick Returns in Rotating Shift Work: A Diary Study. J Occup Environ Med 2017 Jun;59(6):522–7. 10.1097/JOM.0000000000001006 28437294

[10] Åkerstedt T, Perski A, Kecklund G. Sleep, Occupational Stress, and Burnout. In: Kryger MH, Roth T, Dement WC, editors. Principles and practice of sleep medicine. Sixth edition. Philadelphia, PA: Elsevier; 2017.

[11] Kecklund G, Axelsson J. Health consequences of shift work and insufficient sleep. BMJ 2016 Nov;355:i5210. 10.1136/bmj.i5210 27803010

[12] Epstein M, Söderström M, Jirwe M, Tucker P, Dahlgren A. Sleep and fatigue in newly graduated nurses-Experiences and strategies for handling shiftwork. J Clin Nurs 2020 Jan;29(1-2):184–94. 10.1111/jocn.15076 31609523

[13] van de Ven HA, Hulsegge G, Zoomer T, de Korte EM, Burdorf A, Oude Hengel KM. The acute effects of working time patterns on fatigue and sleep quality using daily measurements of 6195 observations among 223 shift workers. Scand J Work Environ Health 2021 Sep;47(6):446–55. 10.5271/sjweh.3964 34029370 PMC8504543

[14] Axelsson J, Åkerstedt T, Kecklund G, Lowden A. Tolerance to shift work-how does it relate to sleep and wakefulness? Int Arch Occup Environ Health 2004 Feb;77(2):121–9. 10.1007/s00420-003-0482-1 14610678

[15] Härmä M, Karhula K, Ropponen A, Puttonen S, Koskinen A, Ojajärvi A et al. Association of changes in work shifts and shift intensity with change in fatigue and disturbed sleep: a within-subject study. Scand J Work Environ Health 2018 Jul;44(4):394–402. 10.5271/sjweh.3730 29641837

[16] Sonnentag S. The recovery paradox: portraying the complex interplay between job stressors, lack of recovery, and poor well-being. Res Organ Behav 2018;17: 10.1016/j.riob.2018.11.002

[17] Sonnentag S, Venz L, Casper A. Advances in recovery research: what have we learned? What should be done next? J Occup Health Psychol 2017 Jul;22(3):365–80. 10.1037/ocp0000079 28358572

[18] Vedaa Ø, Pallesen S, Waage S, Bjorvatn B, Sivertsen B, Erevik E et al. Short rest between shift intervals increases the risk of sick leave: a prospective registry study. Occup Environ Med 2017 Jul;74(7):496–501. 10.1136/oemed-2016-103920 27827302

[19] Nielsen HB, Hansen ÅM, Conway SH, Dyreborg J, Hansen J, Kolstad HA et al. Short time between shifts and risk of injury among Danish hospital workers: a register-based cohort study. Scand J Work Environ Health 2019 Mar;45(2):166–73. 10.5271/sjweh.3770 30264848

[20] Trinkoff AM, Le R, Geiger-Brown J, Lipscomb J. Work schedule, needle use, and needlestick injuries among registered nurses. Infect Control Hosp Epidemiol 2007 Feb;28(2):156–64. 10.1086/510785 17265396

[21] Geurts SA, Sonnentag S. Recovery as an explanatory mechanism in the relation between acute stress reactions and chronic health impairment. Scand J Work Environ Health 2006 Dec;32(6):482–92. 10.5271/sjweh.1053 17173204

[22] Bambra CL, Whitehead MM, Sowden AJ, Akers J, Petticrew MP. Shifting schedules: the health effects of reorganizing shift work. Am J Prev Med 2008 May;34(5):427–34. 10.1016/j.amepre.2007.12.023 18407011

[23] Dahlgren A, Tucker P, Epstein M, Gustavsson P, Söderström M. Randomised control trial of a proactive intervention supporting recovery in relation to stress and irregular work hours: effects on sleep, burn-out, fatigue and somatic symptoms. Occup Environ Med 2022 Jul;79(7):460–8. 10.1136/oemed-2021-107789 35074887 PMC9209685

[24] Kjellberg A, Iwanowski S. Stress/energi-formuläret: utveckling av en metod för skattning av sinnesstämning i arbetet. Solna: Arbetsmiljöinstitutet. Report No. 1989;1989:26.

[25] Dahlgren A, Kecklund G, Åkerstedt T. Different levels of work-related stress and the effects on sleep, fatigue and cortisol. Scand J Work Environ Health 2005 Aug;31(4):277–85. 10.5271/sjweh.883 16161710

[26] Akerstedt T, Gillberg M. Subjective and objective sleepiness in the active individual. Int J Neurosci 1990 May;52(1-2):29–37. 10.3109/00207459008994241 2265922

[27] Keklund G, Åkerstedt T. Objective components of individual differences in subjective sleep quality. J Sleep Res 1997 Dec;6(4):217–20. 10.1111/j.1365-2869.1997.00217.x 9493520

[28] Barr DJ, Levy R, Scheepers C, Tily HJ. Random effects structure for confirmatory hypothesis testing: keep it maximal. J Mem Lang 2013 Apr;68(3):255–78. 10.1016/j.jml.2012.11.001 24403724 PMC3881361

[29] Brysbaert M, Stevens M. Power Analysis and Effect Size in Mixed Effects Models: A Tutorial. J Cogn 2018 Jan;1(1):9. 10.5334/joc.10 31517183 PMC6646942

[30] Bates D, Maechler M, Bolker B, Walker S. Fitting Linear Mixed-Effects Models Using lme4. J Stat Softw 2015;67(1):1–48. 10.18637/jss.v067.i01

[31] Yin J, Jin X, Shan Z, Li S, Huang H, Li P et al. Relationship of Sleep Duration With All-Cause Mortality and Cardiovascular Events: A Systematic Review and Dose-Response Meta-Analysis of Prospective Cohort Studies. J Am Heart Assoc 2017 Sep;6(9):e005947. 10.1161/JAHA.117.005947 28889101 PMC5634263

[32] Åkerstedt T, Anund A, Axelsson J, Kecklund G. Subjective sleepiness is a sensitive indicator of insufficient sleep and impaired waking function. J Sleep Res 2014 Jun;23(3):240–52. 10.1111/jsr.12158 24750198

[33] Åkerstedt T, Folkard S. The three-process model of alertness and its extension to performance, sleep latency, and sleep length. Chronobiol Int 1997 Mar;14(2):115–23. 10.3109/07420529709001149 9095372

[34] Åkerstedt T, Nilsson PM, Kecklund G. Sleep and recovery. In: Sonnentag S, Perrewé PL, Ganster DC, editors. Current Perspectives on Job-Stress Recovery [Internet]. Emerald Group Publishing Limited; 2009. p. 205–47. (Research in Occupational Stress and Well Being; vol. 7). Available from: 10.1108/S1479-3555(2009)0000007009 10.1108/S1479-3555(2009)0000007009

[35] Costa G, Anelli MM, Castellini G, Fustinoni S, Neri L. Stress and sleep in nurses employed in “3 × 8” and “2 × 12” fast rotating shift schedules. Chronobiol Int 2014 Dec;31(10):1169–78. 10.3109/07420528.2014.957309 25216205

