# Supplementary material for: Quick returns, sleep, sleepiness and stress – An intra-individual field study on objective sleep and diary data
Source: Scand J Work Environ Health. 2024 Aug 30;50(6):466–74. doi: 10.5271/sjweh.4175 (PMC11393759; doi:10.5271/sjweh.4175)
Supplement: Supplementary material [file SJWEH-50-466-S001.pdf]

Quick returns, sleep, sleepiness and stress – An intra-individual field study on objective sleep data and diary data[1]

by Kristin Öster, doctoral student,[2] Philip Tucker, PhD, Marie Söderström, PhD, Anna Dahlgren, PhD

1. Supplementary material
2. Kristin Öster, Doctoral student, Karolinska Institutet, Department of Clinical Neuroscience, Division of Psychology, Nobels väg 9, S-171 65 Solna, Sweden. [E-mail: kristin.oster@ki.se]

Model output & sensitivity analyses

Below reads the model output for all original models, together with the results from the sensitivity analyses.

For all outcomes, sensitivity tests of unpaired t-tests were performed, using only data from the second measurement day. The purpose of these analyses was to check sensitivity to order effects, as day-day transitions tended to follow directly after quick returns (evening-day-day). As a result, there could be nonlinear order effects or non linear interactions with the order of shifts, that we couldn't control for in the models. In the sensitivity analysis, the degree of deviance between estimates are of interest but not the p-values.

For stress and sleepiness during work hours, we also conducted sensitivity tests on the pre-registered operationalizations. We checked the degree of deviance in estimates from a strict operationalization including only data from work hours, to the operationalization used in the analysis which included ratings 15 minutes prior to beginning work and 15 minutes after ending ones work. In the strict operationalization, we would only include ratings at 10 and 13 for a person working 07.15-15.45. In the

pre-registered operationalization, the ratings at 07 and 16 are also included in the analysis.

For sleep length, objective (Fragmentation index) and subjective (KSD-SQI) sleep quality - the pre-registered plan was to perform paired sample t-test. To control for order effects, mlm-models were analyzed instead. Here, the results from paired t-tests are also presented to show how they potentially differ.

Stress during work

Original model output:

```
Linear mixed model fit by REML. t-tests use Satterthwaite's method [
lmerModLmerTest]
Formula: stress_work ~ shift + tid + order + (shift + order | id)
   Data: d_stress

REML criterion at convergence: 3021.1

Scaled residuals: 
     Min       1Q   Median       3Q      Max 
-2.91789 -0.58570 -0.03652  0.58197  3.14185 

Random effects:
 Groups   Name        Variance Std.Dev. Corr       
 id       (Intercept) 2.3084   1.5193              
          shiftQR     1.4642   1.2100   -0.55      
          order       0.1629   0.4036   -0.34 -0.09
 Residual             1.7444   1.3208              
Number of obs: 799, groups:  id, 89

Fixed effects:
             Estimate Std. Error        df t value Pr(>|t|)    
(Intercept)   3.70440    0.22105  77.85440  16.758  < 2e-16 ***
shiftQR       0.26101    0.17853  67.79905   1.462   0.1484    
tid10         0.57132    0.12406 563.79564   4.605 5.10e-06 ***
tid13         0.81321    0.12398 565.32958   6.559 1.22e-10 ***
tid16         0.39585    0.16907 603.88289   2.341   0.0195 *  
order         0.21175    0.08304  34.52508   2.550   0.0154 *  
---
Signif. codes:  0 '***' 0.001 '**' 0.01 '*' 0.05 '.' 0.1 ' ' 1

Correlation of Fixed Effects:
        (Intr) shftQR tid10  tid13  tid16 
shiftQR -0.595                            
tid10   -0.303  0.008                     
tid13   -0.308  0.013  0.530              
```

```
tid16   -0.225  0.020  0.388  0.391
order   -0.515  0.213  0.008  0.011 -0.006
```

Sensitivity analysis, unpaired t-test

```
 Welch Two Sample t-test

data:  qr and dd
t = 0.91085, df = 56.818, p-value = 0.3662
alternative hypothesis: true difference in means is not equal to 0
95 percent confidence interval:
 -0.4016352  1.0717984
sample estimates:
mean of x mean of y 
 4.428030  4.092949
```

The estimated difference of the unpaired t-test (diff = 0.34) is close to the estimated effect of shift in the mlm-model ($\beta = 0.26$).

Sensitivity analysis of the operationalization of stress at work

```
Linear mixed model fit by REML. t-tests use Satterthwaite's method [
lmerModLmerTest]
Formula: stressw ~ shift + order + tid + (shift + order | id)
   Data: dtib

REML criterion at convergence: 2959.7

Scaled residuals: 
     Min       1Q   Median       3Q      Max 
-2.90064 -0.58177 -0.03499  0.57732  3.12991 

Random effects:
 Groups   Name        Variance Std.Dev. Corr       
 id       (Intercept) 2.3401   1.5298              
          shiftQR     1.4739   1.2140   -0.57      
          order       0.1497   0.3869   -0.35 -0.04
 Residual             1.7584   1.3260              
Number of obs: 781, groups:  id, 89

Fixed effects:
            Estimate Std. Error       df t value Pr(>|t|)    
(Intercept)   3.6851     0.2230  78.7652  16.523  < 2e-16 ***
shiftQR       0.2858     0.1794  68.1471   1.593  0.11581    
order         0.2091     0.0825  35.3231   2.535  0.01584 *  
tid10         0.5867     0.1254 549.9513   4.680 3.62e-06 ***
tid13         0.8231     0.1251 551.1589   6.578 1.11e-10 ***
tid16         0.5049     0.1811 593.0499   2.788  0.00547 ** 
---
Signif. codes:  0 '***' 0.001 '**' 0.01 '*' 0.05 '.' 0.1 ' ' 1

Correlation of Fixed Effects:
```

```
       (Intr) shftQR order  tid10  tid13
shiftQR -0.604
order   -0.519  0.238
tid10   -0.307  0.009  0.010
tid13   -0.311  0.014  0.011  0.535
tid16   -0.216  0.024 -0.007  0.369  0.372
```

The estimated effect of shift in the model with the strict operationalization ($\beta = 0.29$) does not deviate from that of the pre-registered operationalization in the original model ($\beta = 0.26$).

Sleepiness during work

Original model output:

```
Linear mixed model fit by REML. t-tests use Satterthwaite's method [
lmerModLmerTest]
Formula: KSS_work ~ shift + tid + order + (shift + order | id)
   Data: d_KSSwork

REML criterion at convergence: 2899.1

Scaled residuals:
    Min      1Q  Median      3Q     Max
-2.9316 -0.6066 -0.0899  0.6317  3.2473

Random effects:
 Groups   Name        Variance Std.Dev. Corr
 id       (Intercept) 2.67123  1.6344
          shiftQR     0.58547  0.7652   -0.38
          order       0.04274  0.2067   -0.84  0.05
 Residual             1.55736  1.2479
Number of obs: 810, groups:  id, 90

Fixed effects:
            Estimate Std. Error        df t value Pr(>|t|)
(Intercept)  4.59868    0.27246  67.02732  16.878  < 2e-16 ***
shiftQR      0.45039    0.13163  69.05962   3.422  0.00105 **
tid10       -0.92996    0.11634 621.99887  -7.994 6.39e-15 ***
tid13       -0.63573    0.11636 624.11652  -5.463 6.75e-08 ***
tid16        0.24345    0.15629 662.46565   1.558  0.11979
order        0.08464    0.05650  42.57929   1.498  0.14155
---
Signif. codes:  0 '***' 0.001 '**' 0.01 '*' 0.05 '.' 0.1 ' ' 1

Correlation of Fixed Effects:
       (Intr) shftQR tid10  tid13  tid16
shiftQR -0.489
tid10   -0.234  0.009
tid13   -0.236  0.012  0.528
```

```
tid16   -0.167  0.020  0.392  0.394
order   -0.832  0.290  0.010  0.011 -0.008
```

Sensitivity analysis, unpaired t-test

```
 Welch Two Sample t-test

data:  qr and dd
t = 1.6364, df = 49.549, p-value = 0.1081
alternative hypothesis: true difference in means is not equal to 0
95 percent confidence interval:
 -0.1342752  1.3137624
sample estimates:
mean of x mean of y
 4.833333  4.243590
```

The estimated difference of the unpaired t-test (diff = 0.59) is close to the estimated effect of shift in the mlm-model ($\beta = 0.45$).

Sensitivity analysis of operationalisation

```
Linear mixed model fit by REML. t-tests use Satterthwaite's method [
lmerModLmerTest]
Formula: kssw ~ shift + tid + order + (shift | id)
   Data: dtib

REML criterion at convergence: 2843.9

Scaled residuals:
    Min      1Q  Median      3Q     Max
-2.8546 -0.6157 -0.0920  0.6186  3.2796

Random effects:
 Groups   Name        Variance Std.Dev. Corr
 id       (Intercept) 1.3182   1.1481
          shiftQR     0.6671   0.8168   -0.43
 Residual             1.5722   1.2539
Number of obs: 792, groups:  id, 90

Fixed effects:
             Estimate Std. Error        df t value Pr(>|t|)
(Intercept)   4.66408    0.24472 338.76148  19.059  < 2e-16 ***
shiftQR       0.41462    0.13399  99.20887   3.094  0.00256 **
tid10        -0.91859    0.11758 619.56219  -7.813 2.41e-14 ***
tid13        -0.62473    0.11747 621.33661  -5.318 1.46e-07 ***
tid16         0.18640    0.16719 657.07006   1.115  0.26531
order         0.06992    0.05308 471.10156   1.317  0.18838
---
```

```
Signif. codes:  0 '***' 0.001 '**' 0.01 '*' 0.05 '.' 0.1 ' ' 1

Correlation of Fixed Effects:
        (Intr) shftQR tid10  tid13  tid16
shiftQR -0.507
tid10   -0.266  0.010
tid13   -0.268  0.014  0.533
tid16   -0.184  0.026  0.374  0.376
order   -0.767  0.295  0.012  0.013 -0.005
```

The estimated effect of shift in the model with the strict operationalization ($\beta = 0.41$) does not deviate from that of the pre-registered operationalization ($\beta = 0.45$).

Sleepiness during leisure.

Original model output:

```
Linear mixed model fit by REML. t-tests use Satterthwaite's method [
lmerModLmerTest]
Formula: KSS_leisure ~ shift + tid + order + (shift | id)
   Data: d_leisure

REML criterion at convergence: 2304.1

Scaled residuals:
     Min       1Q   Median       3Q      Max
-2.92479 -0.57623  0.06721  0.61024  2.45052

Random effects:
 Groups   Name        Variance Std.Dev. Corr
 id       (Intercept) 1.057    1.0283
          shiftQR     0.510    0.7142   -0.17
 Residual             2.427    1.5579
Number of obs: 579, groups:  id, 90

Fixed effects:
             Estimate Std. Error        df t value Pr(>|t|)
(Intercept)   5.21362    0.20713 226.73541  25.171  < 2e-16 ***
shiftQR       0.35754    0.16457 104.84680   2.173   0.0321 *
tid19        -0.16567    0.16334 443.58219  -1.014   0.3110
tid22         1.13348    0.17304 456.74377   6.550 1.55e-10 ***
order         0.05864    0.06822 325.84751   0.860   0.3907
---
Signif. codes:  0 '***' 0.001 '**' 0.01 '*' 0.05 '.' 0.1 ' ' 1

Correlation of Fixed Effects:
        (Intr) shftQR tid19  tid22
shiftQR -0.467
tid19   -0.473  0.010
tid22   -0.438  0.004  0.571
order   -0.482  0.305 -0.012 -0.042
```

Sensitivity analysis, unpaired t-test

```
 Welch Two Sample t-test

data:  qr and dd
t = 0.49484, df = 57.908, p-value = 0.6226
alternative hypothesis: true difference in means is not equal to 0
95 percent confidence interval:
 -0.5548357  0.9192232
sample estimates:
mean of x mean of y
 5.829630  5.647436
```

The estimated difference of the unpaired t-test (diff = 0.18) is half the size of the estimated effect of shift in the mlm-model ($\beta = 0.36$). The result indicates that there may be confounding due to the order of shift.

Sleepiness - interaction model

Original model output

```
## Linear mixed model fit by REML. t-tests use Satterthwaite's method [
## lmerModLmerTest]
## Formula: KSS_work ~ shift:tid + order + shift + tid + (shift | id)
##          Data: d_H5agg
##
## REML criterion at convergence: 2374.4
##
## Scaled residuals:
##          Min          1Q   Median    3Q          Max
## -2.77473 -0.58053 -0.06905  0.56451  3.00882
##
## Random effects:
##  Groups   Name        Variance Std.Dev. Corr
##  id       (Intercept) 1.3954   1.1813
##           shiftQR     0.7047   0.8394   -0.43
##  Residual             1.2865   1.1342
## Number of obs: 685, groups:  id, 90
##
## Fixed effects:
##          Estimate Std. Error        df t value Pr(>|t|)
## (Intercept)         4.60001        0.31389 198.61467  14.655  < 2e-16 ***
## order      0.08860      0.07300 135.55923  1.214 0.226995
## shiftQR    0.46516      0.20685 376.02132  2.249 0.025101 *
## tid10      -0.86003     0.17799 506.79656  -4.832  1.8e-06 ***
## tid13      -0.66025     0.17791 513.32699  -3.711 0.000229 ***
## tid16      0.22441      0.17795 509.88478  1.261 0.207847
## shiftQR:tid10  -0.18780  0.24714 505.93654  -0.760 0.447690
## shiftQR:tid13   0.07169  0.24703 509.67449  0.290 0.771777
```

```
## shiftQR:tid16   0.18499  0.25057 510.15429   0.738 0.460675
## ---
## Signif. codes:  0 '***' 0.001 '**' 0.01 '*' 0.05 '.' 0.1 ' ' 1
##
## Correlation of Fixed Effects:
##              (Intr) order  shftQR tid10  tid13  tid16  sQR:10 sQR:13
## order        -0.820
## shiftQR      -0.549  0.266
## tid10        -0.300  0.004  0.451
## tid13        -0.308  0.010  0.458  0.523
## tid16        -0.305  0.008  0.454  0.521  0.528
## shftQR:td10  0.216 -0.004 -0.620 -0.720 -0.376 -0.375
## shftQR:td13  0.222 -0.008 -0.625 -0.377 -0.720 -0.380  0.518
## shftQR:td16  0.214 -0.003 -0.614 -0.370 -0.375 -0.710  0.511  0.514
```

Fragmentation index

Original model output:

```
Linear mixed model fit by REML. t-tests use Satterthwaite's method [
lmerModLmerTest]
Formula: fragmentation_index ~ shift + order + (shift | id)
   Data: d_wide

REML criterion at convergence: 1710.6

Scaled residuals:
     Min       1Q   Median       3Q      Max
-1.90113 -0.49427 -0.01652  0.47316  2.66403

Random effects:
 Groups   Name        Variance Std.Dev. Corr
 id       (Intercept) 34.86    5.904
          shiftQR     26.32    5.130    -0.19
 Residual             40.08    6.331
Number of obs: 242, groups:  id, 87

Fixed effects:
            Estimate Std. Error       df t value Pr(>|t|)
(Intercept)  23.9484     1.7298 200.1436  13.845   <2e-16 ***
shiftQR       1.7213     1.0539  95.1611   1.633    0.106
order        -1.0743     0.4288 203.4932  -2.505    0.013 *
---
Signif. codes:  0 '***' 0.001 '**' 0.01 '*' 0.05 '.' 0.1 ' ' 1

Correlation of Fixed Effects:
        (Intr) shftQR
shiftQR -0.477
order   -0.866  0.288
```

Sensitivity analysis, unpaired t-test

```
 Welch Two Sample t-test
```

```
data:  qr and dd
t = -0.84092, df = 52.357, p-value = 0.4042
alternative hypothesis: true difference in means is not equal to 0
95 percent confidence interval:
 -6.905903  2.826655
sample estimates:
mean of x mean of y
 21.84884  23.88846
```

The estimated difference from the sensitivity analysis is in the opposite direction to that of the mlm-model (diff = -2.04 vs. $\beta = 1.72$).

Sensitivity analysis, paired t-test

```
 Paired t-test

data:  qr_FI and dd_FI
t = 2.304, df = 86, p-value = 0.02364
alternative hypothesis: true difference in means is not equal to 0
95 percent confidence interval:
 0.3150389 4.2784477
sample estimates:
mean of the differences
              2.296743
```

The paired t-test results in a significant difference of 2.3 percentage points more fragmented sleep during quick returns compared to day-day transitions (p = 0.024). This estimate does not, however, control for the confounding effect of order. The mlm-model (presented above) indicates that when order is inserted into the model, the effect of shift is no longer significant. As you can see in the model output, there is also a correlation between the parameters shift and order, which indicates that we cannot fully discern the effect of shift from that of order and vice versa. Thus, the data is inconclusive but more than not indicates that the difference we see here is due to the number of consecutive workdays (=order) rather than an effect of shift.

KSD-SQI

Original model output:

```
Linear mixed model fit by REML. t-tests use Satterthwaite's method [
lmerModLmerTest]
```

```
Formula: KSD_SQI ~ shift + order + (shift | id)
   Data: d_wide

REML criterion at convergence: 593.3

Scaled residuals: 
    Min      1Q  Median      3Q     Max 
-2.7398 -0.5441  0.1204  0.6321  1.7429 

Random effects:
 Groups   Name        Variance Std.Dev. Corr 
 id       (Intercept) 0.1057   0.3250        
          shiftQR     0.2492   0.4992   -0.05
 Residual             0.4286   0.6547        
Number of obs: 250, groups:  id, 90

Fixed effects:
             Estimate Std. Error        df t value Pr(>|t|)    
(Intercept)   4.20498    0.15409 195.20574  27.289  < 2e-16 ***
shiftQR      -0.49145    0.10400  99.38662  -4.725 7.57e-06 ***
order        -0.01577    0.03960 217.20390  -0.398    0.691    
---
Signif. codes:  0 '***' 0.001 '**' 0.01 '*' 0.05 '.' 0.1 ' ' 1

Correlation of Fixed Effects:
        (Intr) shftQR
shiftQR -0.468       
order   -0.897  0.271
```

Sensitivity analysis, unpaired t-test

```
 Welch Two Sample t-test

data:  qr and dd
t = -2.6436, df = 57.708, p-value = 0.01055
alternative hypothesis: true difference in means is not equal to 0
95 percent confidence interval:
 -0.8514592 -0.1176044
sample estimates:
mean of x mean of y 
 3.717391  4.201923 
```

The estimated difference of the unpaired t-test (diff = -0.48) is essentially equal to the estimated effect of shift in the mlm-model (β = -0.49).

Sensitivity analysis, paired t-test

```
 Paired t-test

data:  qr_ksd and dd_ksd
t = -4.8087, df = 88, p-value = 6.228e-06
```

```
alternative hypothesis: true difference in means is not equal to 0
95 percent confidence interval:
 -0.6576735 -0.2730382
sample estimates:
mean of the differences
           -0.4653558
```

The paired t-test (diff = -0.47) and the mlm model (β = -0.49) results in an essentially equal estimated difference in sleep quality between conditions.

Sleep length

Results from original model (paired t-test)

```
 Paired t-test

data:  qr_sleep and dd_sleep
t = -9.6864, df = 86, p-value = 1.972e-15
alternative hypothesis: true difference in means is not equal to 0
95 percent confidence interval:
 -1.2304338 -0.8113925
sample estimates:
mean of the differences
            -1.020913
```

Sensitivity analysis, unpaired t-test

```
 Welch Two Sample t-test

data:  qr and dd
t = -6.2532, df = 43.612, p-value = 1.481e-07
alternative hypothesis: true difference in means is not equal to 0
95 percent confidence interval:
 -1.6665295 -0.8539833
sample estimates:
mean of x mean of y
 6.000000  7.260256
```

The unpaired t-test results in an observed difference in sleep length (diff = -1.26) of the same order (slightly larger) of the paired t-test (diff = -1.02).

Secondary outcomes

Sleep efficiency (%)

Results from original model (paired t-test)

```
 Paired t-test

data:  qr_perc and dd_perc
t = -2.3051, df = 86, p-value = 0.02357
alternative hypothesis: true difference in means is not equal to 0
95 percent confidence interval:
 -1.443540 -0.106652
sample estimates:
mean of the differences
          -0.7750958
```

Sensitivity test (unpaired t-test)

```
 Welch Two Sample t-test

data:  qr and dd
t = 1.2274, df = 45.638, p-value = 0.226
alternative hypothesis: true difference in means is not equal to 0
95 percent confidence interval:
 -0.8086302  3.3345693
sample estimates:
mean of x mean of y
 89.68605  88.42308
```

The unpaired t-test gives an estimate in the opposite direction (diff = 1.26 percentage points) of that of the paired t-test (diff = -0.78 percentage points). Both estimates are small, and the effect observed in the main analysis may be due to chance.

Anxious at bedtime

Results from original model (paired t-test)

```
 Paired t-test

data:  qr_anx_bed and dd_anx_bed
t = -2.4736, df = 89, p-value = 0.01527
alternative hypothesis: true difference in means is not equal to 0
95 percent confidence interval:
 -0.68791758 -0.07504538
sample estimates:
mean of the differences
          -0.3814815
```

Sensitivity test (unpaired t-test)

```
 Welch Two Sample t-test

data:  qr and dd
t = -1.512, df = 55.961, p-value = 0.1362
alternative hypothesis: true difference in means is not equal to 0
95 percent confidence interval:
 -1.0380480  0.1450714
sample estimates:
mean of x mean of y
 3.630435  4.076923
```

The observed difference in the unpaired t-test (diff = -0.45) is close to that of the paired t-test in the main analysis (diff = -0.38).

Feeling rested

Results from original model (paired t-test)

```
 Paired t-test

data:  qr_rested and dd_rested
t = -4.5996, df = 89, p-value = 1.395e-05
alternative hypothesis: true difference in means is not equal to 0
95 percent confidence interval:
 -0.7716814 -0.3060963
sample estimates:
mean of the differences
            -0.5388889
```

Sensitivity test (unpaired t-test)

```
 Welch Two Sample t-test

data:  qr and dd
t = -3.2266, df = 40.818, p-value = 0.002471
alternative hypothesis: true difference in means is not equal to 0
95 percent confidence interval:
 -1.2616418 -0.2901977
sample estimates:
mean of x mean of y
 2.108696  2.884615
```

The observed difference in the unpaired t-test in the sensitivity analysis (diff = -0.78) is 50 % larger compared to the observed difference in the paired t-test of the main analysis (diff = -0.54). In the main analysis, we have not controlled for order effects. It

could be that some of the difference in feeling rested is masked by the number of consecutive work days (day-day transitions usually follow after quick returns).

Feeling tense during the day

Results from original model (paired t-test)

```
 Paired t-test

data:  qr_tense and dd_tense
t = 0.11478, df = 89, p-value = 0.9089
alternative hypothesis: true difference in means is not equal to 0
95 percent confidence interval:
 -0.2718567  0.3051901
sample estimates:
mean of the differences
          0.01666667
```

Sensitivity test (unpaired t-test)

```
 Welch Two Sample t-test

data:  qr and dd
t = 1.6007, df = 58.197, p-value = 0.1149
alternative hypothesis: true difference in means is not equal to 0
95 percent confidence interval:
 -0.1155965  1.0386735
sample estimates:
mean of x mean of y
 2.500000  2.038462
```

Whereas the paired t-test of the main analysis indicates no difference between conditions (diff = 0.02), the unpaired t-test does so (diff = 0.46) although not significant. The results are inconclusive.

Ability to turn off work - during the first day of either transition (evening-day // day-day)

Results from paired t-test

```
##
##  Paired t-test
##
## data:  qr_detach_lagged and dd_detach_lagged
## t = 1.5385, df = 89, p-value = 0.1275
## alternative hypothesis: true difference in means is not equal to 0
## 95 percent confidence interval:
```

```
## -0.0631554 0.4964887
## sample estimates:
## mean of the differences
##           0.2166667
```

Sensitivity test (unpaired t-test)

```
##
## Welch Two Sample t-test
##
## data:  qr and dd
## t = 1.1757, df = 49.748, p-value = 0.2453
## alternative hypothesis: true difference in means is not equal to 0
## 95 percent confidence interval:
## -0.2808501 1.0734922
## sample estimates:
## mean of x mean of y
## 2.434783 2.038462
```

The observed difference in the unpaired t-test in the sensitivity analysis (diff = 0.4) is more than twice the size compared to the observed difference in the paired t-test of the main analysis (diff = 0.22). Both are non-significant, but it could be that the difference in psychological detachment is masked by the order in which shifts appear.

Ability to turn off work - during the second day of either transition (evening-day // day-day)

Results from original model (paired t-test)

```
 Paired t-test

data:  qr_detach and dd_detach
t = 1.5709, df = 89, p-value = 0.1198
alternative hypothesis: true difference in means is not equal to 0
95 percent confidence interval:
 -0.05543139 0.47394991
sample estimates:
mean of the differences
          0.2092593
```

Sensitivity test (unpaired t-test)

```
 Welch Two Sample t-test

data:  qr and dd
t = 1.6231, df = 59.859, p-value = 0.1098
alternative hypothesis: true difference in means is not equal to 0
95 percent confidence interval:
 -0.1180365 1.1336886
```

```
sample estimates:
mean of x mean of y
 2.347826  1.840000
```

Again, the observed difference in the unpaired t-test in the sensitivity analysis (diff = 0.51) is more than twice the size compared to the observed difference in the paired t-test of the main analysis (diff = 0.21). Both are non-significant, but it could be that the difference in psychological detachment is masked by the order in which shifts appear.
